# Patient Satisfaction with Oncological Care during the SARS-CoV-2 Virus Pandemic

**DOI:** 10.3390/ijerph18084122

**Published:** 2021-04-13

**Authors:** Magdalena Konieczny, Elżbieta Cipora, Jolanta Sawicka, Andrzej Fal

**Affiliations:** 1 Medical Institute, The Jan Grodek State University in Sanok, 38-500 Sanok, Poland; elacipora@interia.pl (E.C.); sawickaj@poczta.fm (J.S.); 2 Faculty of Medicine, Collegium Medicum, Cardinal Stefan Wyszyński University, 01-938 Warsaw, Poland; amfal@wp.pl

**Keywords:** pandemic, COVID-19, cancer, cancer treatment, satisfaction, care

## Abstract

Recently, the outbreak of the SARS-CoV-2 virus and the COVID-19 pandemic significantly affected the health situation of the entire society and necessitated reorganization of health care including oncology. The objective of this study was to examine the perception of medical services by cancer patients during the pandemic and to identify the key elements influencing the level of satisfaction with oncological care. Of note, 394 patients diagnosed with cancer treated in inpatient oncology wards participated in the study (Poland). The diagnostic survey method was used. A survey questionnaire developed by the authors was used and validated the EORTC IN-PATSAT32 questionnaire. The calculations were made in Statistica 10.0 (Statsoft; 2011, Dell Inc., Round Rock, TX, USA). The average general level of satisfaction with oncological care in the study group was 80.77 out of a total score of 100, representing the highest level of satisfaction. Levels of satisfaction varied according to time since diagnosis (longer time—greater satisfaction) and were lower where treatment was delayed or perceived as disorganised. Nearly half of the respondents felt the threat of the SARS-CoV-2 infection, despite the fact that most of them believed that the hospital was well prepared to diagnose and treat cancer patients during the COVID-19 pandemic. Convincing patients about the proper preparation of health care for diagnostics and therapy is an important element influencing patient satisfaction with oncological care.

## 1. Introduction

Severe acute respiratory syndrome coronavirus-2 (SARS-CoV-2) and the resulting disease COVID-19 were detected in Wuhan in late 2019. By the end of March 2021, almost 132 million cases of COVID-19 infections were confirmed worldwide [1]. In Poland, the first case of the disease was diagnosed on 4 March 2020, and by the end of March, there were 2,321,717 cases of COVID-19 infections, of which 53,045 were fatal [2,3,4].

The COVID-19 pandemic has significantly changed all spheres of human life and made it necessary to reorganize medical care around the world. The epidemiological situation and the resulting health needs influenced the need to organize medical care not only in terms of treatment of COVID-19 patients but also the method of providing professional care for cancer patients, who in every society constitute a significant percentage of people in need of therapy [5,6].

In Poland, approximately 450,000 people are living with cancer and the number is constantly growing. Compared to most OECD countries (Organization for Economic Co-operation and Development), Poland has lower rates of cancer morbidity, but at the same time higher cancer mortality. Per 100,000, statistically 237 people die, while the average for OECD countries is 201 deaths [7]. For many reasons, cancer patients constitute a special group of patients, among others, they have significantly reduced immunity and therefore they are exposed to viral infections to a greater extent than other patients. It has been shown that people with active cancer have a significantly higher risk of COVID-19 [8,9].

In addition, the morbidity of most cancers increases with age, as a consequence, the largest group of patients are elderly patients, which additionally makes these people more susceptible to SARS-CoV-2 infections and at risk of complications and death in the course of COVID-19 [10]. Another factor that makes cancer patients susceptible to infections, including SARS-CoV-2, is systemic antitumor therapy (mainly chemotherapy) [11,12]. The mere necessity to conduct anticancer therapy in a hospital setting is a factor that increases the risk of the SARS-CoV-2 infection [13].

Summing up—due to numerous factors, during the COVID-19 pandemic, cancer patients are particularly vulnerable to infections and a more severe course of the disease, with a greater number of complications and a higher risk of death.

As a result, it is a group of patients who require special care during this period. Additionally, the deteriorating situation of Polish cancer patients, resulting from the organization and level of funding, makes providing care for cancer patients during the pandemic very challenging [14,15,16]. It should be emphasised that oncological diagnostics and treatment during the pandemic is difficult. Numerous clinics suspended their activities, and contact with a general practitioner was limited to teleconsultation. Maluchnik et al. [2] reported that, in 2020, the number of preliminary oncological diagnoses in Poland decreased by 31% compared to 2019. There was also a decrease in extended diagnostic procedures (by 25%) and oncological consultations (by 19%) [2].

Unfortunately, the COVID-19 pandemic also causes or intensifies a sense of anxiety, and intensifies depression, which is most often more symptomatic in cancer patients on a daily basis [17]. In addition to the significant impact of this factor on the quality of life and well-being of patients, it may also result in poor perception of the changes introduced in oncological care [18].

Numerous recommendations have been introduced at oncological centres in order to limit the transmission of the virus, and multiple expert groups have published guidelines on care for cancer patients during the pandemic [19]. Apart from assessing the effectiveness of such recommendations, their acceptance by patients is very important. So far, there are few research results that take into account patients’ assessment of the changes and restrictions introduced.

The objective of this study was to examined the perception of medical services by cancer patients during the pandemic and to identify the key elements influencing the level of satisfaction with oncological care. The information obtained will improve the quality and direct targeting of services to cancer patients during the pandemic.

## 2. Materials and Methods

### 2.1. Study Participants, Data Collection and Analysis

The qualitative research was carried out by Fr. B. Markiewicz’s Podkarpackie Oncology Centre in Brzozów (Brzozów, Poland) over a period of three months—from July to September 2020. The selection of the centre to carry out the study was intentional. The Podkarpacie Oncology Centre is a leading cancer treatment facility in southeastern Poland. In 2020, oncological care due to the diagnosis of malignant cancer was provided to nearly 15,000 patients, of which 4013 patients were hospitalized. The study was approved by the director of the hospital. The study was also approved by the Bioethics Committee (No. 03/2020; as of 2 July 2020). The criteria for inclusion in the study were: malignant cancer diagnosis, inpatient treatment (hospitalization) for at least 3 days at the Podkarpackie Cancer Centre in oncological wards (i.e., in the wards of: clinical oncology, oncological surgery, oncological haematology, oncological orthopaedics, radiotherapy), expressed consent to participate in the study, age over 18. Patients who did not consent to participate in the study and those who were not able to complete the questionnaire on their own due to their health were excluded from the study. In the group of patients who met the assumed inclusion and exclusion criteria, the principle of random sampling was applied. All patients were informed about the objective of the study, ensured anonymity and voluntary participation in it. Incomplete questionnaires were rejected. A total of 394 patients participated in the study. The response rate was 97.3%; 405 questionnaires were distributed, 11 questionnaires were rejected as they were incomplete. The process of distributing and collecting the questionnaires was monitored by the oncological treatment coordinator of the Podkarpackie Cancer Centre. The questionnaires were completed by patients on an ongoing basis.

The calculations were made using the Statistica 10.0 package (Statsoft; 2011, Dell Inc., Round Rock, TX, USA). Compliance with the normal distribution was tested with the Shapiro–Wilk test. The assumption of homogeneity of variance was checked using Levene’s test. Due to the fact that there was a large study sample (*N* = 394), research hypotheses were verified using parametric methods. Comparisons for the two groups were made with the Student’s *t*-test for independent variables. The Cochran-Cox test was used when the assumption of equal variance was not met. Comparisons for more than two groups were performed using one-way analysis of variance or Welch’s test. HSD Tukey’s test for unequal numbers was used for multiple comparisons. The significance level of α = 0.05 was assumed, the results were considered statistically significant when the calculated test probability p met the condition of *p* ≤ 0.05.

### 2.2. Research Tools—Questionnaires

The studies conducted were aimed at obtaining information on the assessment of oncological care during the COVID-19 pandemic. Participants of the study received a set of questionnaires to be completed, i.e., the proprietary questionnaire (Appendix A) and a standardized tool—EORTC IN-PATSAT 32. The proprietary questionnaire prepared for the study consisted of two parts. The first part concerned sociodemographic data, while the second part contained information on the safety and satisfaction with oncological care during the SARS-CoV-2 virus pandemic.

The EORTC IN-PATSAT32 questionnaire was developed by the European Organization for Research and Treatment of Cancer Quality of Life Questionnaire (EORTC) group and is used to assess patient satisfaction with oncological care. This tool consists of 32 questions assessing the quality of work of doctors and nurses, as well as selected aspects of the organization of oncological care and the hospital environment. The questionnaire is divided into eleven multi-element scales, taking into account technical skills of doctors and nurses, interpersonal skills, providing information, availability, interpersonal skills of other hospital employees, waiting time for the implementation of medical procedures, access to the hospital. In addition, the tool included three single questions on information exchange, comfort in the hospital, and overall satisfaction with care in the hospital. Patients answered the questions by marking one of the five answers: “bad”, “fair”, “good”, “very good”, “excellent”. The obtained results were analysed according to the EORTC guidelines [20]. Patient satisfaction with oncological care was assessed (EORTC IN-PATSAT32) depending on: the date of cancer diagnosis, delays in treatment and patients’ opinions on the preparation of the hospital for diagnostics and treatment during the coronavirus pandemic. Delays in treatment were defined as the time from the scheduled date of treatment to the date when the planned medical procedure was actually carried out.

The raw coefficient was calculated, and then the linear transformation was performed in order to obtain a coefficient (scoring), the value of which ranged from 0 to 100 points both for the scales and for individual questions. A higher score meant a higher level of satisfaction with oncological care.

### 2.3. Limitations

The conducted study had limitations, including the fact that it was conducted in only one oncology centre. In addition, satisfaction with oncological care may have been influenced by other factors that were not analysed in this publication (e.g., type of cancer, advancement, sociodemographic factors). Nevertheless, the use of the validated and commonly accepted EORTC IN-PATSAT32 questionnaire allowed for the determination of conclusions resulting from the study.

## 3. Results

### 3.1. Patient Characteristics

Of note, 394 patients participated in the study; the sociodemographic and medical characteristics of the group are presented in Table 1.

### 3.2. Patient Satisfaction and the Perception of Oncological Care during the COVID-19 Pandemic

The vast majority of respondents (86.04%) were of the opinion that the hospital was adequately prepared for diagnostics and treatment of cancer patients during the coronavirus pandemic (SARS-CoV-2), only a small percentage of patients (1.01) was of a different opinion, and the remaining 12.95% did not have an opinion on this topic. Despite this, 43.91% of patients felt at risk of contracting the virus while in hospital. According to the respondents, the most important activities preceding hospitalization that are important to reduce the risk of SARS-CoV-2 infection include: epidemiological interview (according to 66.77%), temperature measurement (54.82%), health assessment (46.95%). The vast majority of the respondents (96.20%) stated that during their hospitalization, doctors and nurses were equipped with appropriate personal protective equipment. In places such as the waiting room and registration room, the rules of the sanitary regime were respected—in particular, keeping a 2 m distance (76.40%). A detailed list is presented in Table 2.

Most of the patients (72.08%) believed that their treatment was carried out as planned, while 27.92% of the respondents believed that there were delays in the oncological treatment process. Patients expressed different views on the impact of the pandemic on cancer diagnostics and treatment. Most of the respondents stated that cancer diagnostics and treatment were partially disorganized due to the SARS-CoV-2 virus. Detailed data on the patients’ opinions on the organization of cancer diagnostics and treatment during the pandemic are presented in Table 3.

### 3.3. Patient Perception of Quality of Care—EORTC IN—PATSAT 32

The average level of patient satisfaction with oncological care during their hospitalization was 80.77. Patient satisfaction with medical and nursing care and the assessment of other areas of the hospital’s activity are presented in Table 4.

Then, patient satisfaction with oncological care was assessed taking into account the time of cancer diagnosis (Table 5). Patients who were diagnosed with cancer in 2018 and earlier assessed: technical skills of doctors (*p* = 0.007) and their availability (*p* = 0.002), and in the case of nursing care, technical skills (*p* = 0.023), providing information (*p* = 0.004) and availability (*p* = 0.003) compared to patients diagnosed with cancer later. In other areas of activity and services provided by the hospital, higher scale values were also obtained for patients diagnosed with cancer in 2018 and earlier (Table 5). This concerned the following categories: other hospital staff, interpersonal skills and availability of information (*p* = 0.004), hospital availability (*p* = 0.008), information exchange (*p* = 0.001), comfort (*p* = 0.014).

A relationship was found between the occurrence of delays in cancer treatment and patient satisfaction with oncological care. Patients whose treatment was delayed due to the COVID-19 pandemic had lower satisfaction with care compared to those who were treated as planned—without delays (*p* = 0.009). Statistically significant differences were found in the vast majority of the studied areas (Table 6).

People who stated that the spread of the SARS-CoV-2 virus disrupted cancer treatment and diagnostics in Poland had lower satisfaction with oncological care compared to those who were of a different opinion. Statistically significant differences were found in all scales of the EORTC IN-PATSAT 32 questionnaire. Detailed results are presented in Table 7.

## 4. Discussion

The COVID-19 pandemic has disrupted the healthcare system in the world. Access to medical care has been limited. The epidemiological situation resulting from the pandemic has had a significant impact on oncological care. Patients hospitalized due to the need for cancer treatment are a special group at risk of COVID-19 and the severe course of this disease [21].

To our knowledge, this is one of the first studies to assess patient satisfaction with oncological care in Poland during the COVID-19 pandemic. According to these patients, the COVID-19 pandemic has had a significant impact on cancer treatment and diagnostics. The sense of anxiety, which is a common problem and has a negative impact on the quality of life, treatment, satisfaction with oncological care and the outcome of therapy in this group of patients is of particular importance [22,23]. Fear and anxiety about the coronavirus exacerbate this problem. Our studies showed that 43.91% of patients also felt the risk of being infected with SARS-CoV-2 during their hospitalization at an oncological ward.

In the studies conducted by Prajoko and Supit [24], as many as 81% of patients receiving chemotherapy were afraid of the SARS-CoV-2 virus infection during their hospitalization, and 13.6% of the respondents considered discontinuing the treatment. Similarly, studies conducted by Joode et al. [25] showed that cancer patients were concerned about the consequences of the pandemic and its influence on the treatment process. Patients whose treatment was delayed (55%) and those who ceased treatment (62%) were particularly concerned. The authors also showed that 47% of patients feared that they would be infected with SARS-CoV-2 while in hospital, which is consistent with the results of our study (43.91%).

In our study, 86.04% of the patients believed that the hospital is properly prepared for cancer diagnostics and treatment during the SARS-CoV-2 pandemic, and that the hospital uses appropriate procedures before admitting a patient, i.e., epidemiological interview, temperature measurement, etc. The vast majority of the respondents (96.2%) stated that during their hospitalization, doctors and nurses were equipped with appropriate personal protective equipment, and the rules of the sanitary regime were respected, in particular keeping a 2 m distance (76.40%) in the waiting room and registration room. Similarly, in the studies by Prajoko and Supit [24], the vast majority of patients believed that the hospital’s safety policy during the COVID-19 pandemic was appropriate. Therefore, it can be assumed that the application of procedures increases the sense of patient safety and reduces the level of anxiety and stress, although these activities do not provide a 100% guarantee in the prevention of coronavirus transmission.

The overall level of patient satisfaction with oncological care included in the study, as determined by EORTC IN-PATSAT 32, was 80.77. In terms of competences of doctors and nurses, interpersonal skills were rated the lowest, the value of these scales was 74.13 and 79.34, respectively. Hospital accessibility was assessed particularly low (69.83). This value was lower compared to the studies conducted by Sánchez et al. [26], in which the overall level of satisfaction with oncological care of women with breast cancer was 91.4, and the availability of medical personnel was 81 for doctors, and 84.2 for nurses.

Our studies showed that patients diagnosed with cancer in 2020 rated technical skills of doctors and nurses, and their availability lower than those who had previously been diagnosed with cancer. Lower satisfaction with oncological care in this group of patients was also expressed by a lower assessment of information exchange and hospital availability. The lower rating may be related to the fact that cancer diagnostics in patients diagnosed in 2020 took place during the pandemic. The sense of safety of this group has been disturbed not only by the disease but also by the epidemiological situation, the focus of the healthcare system on COVID-19 and the resulting concern about the possibility of proper treatment of other diseases.

Thus, during the COVID-19 pandemic, stress related to cancer diagnosis increased as a result of organizational changes in the healthcare system. Similarly, in their study, Magno et al. [27] showed that increased anxiety among patients with breast cancer awaiting surgery was related to concerns regarding delays in treatment due to the pandemic and greater susceptibility to infection.

In this study, it was found that 27.92% of patients experienced delays in cancer treatment. There was an obvious link between delays in treatment and patient satisfaction with oncological care. Patients who experienced delays in treatment due to the COVID-19 pandemic had lower satisfaction with care compared to those who were treated as planned. Consequently, lower satisfaction with oncological care translated into a lower assessment of competences of doctors and nurses, as well as the hospital’s activity in other areas.

In a study involving 5302 patients conducted in 2020 in the Netherlands, it was found that 30% of the respondents felt a significant impact of the pandemic on the organization and course of cancer treatment. In most cases (52%), the changes were related to the introduction of telephone or online consultations instead of traditional hospital appointments and postponement of therapy—in 16% of the patients in the group waiting for treatment and in 12% of the patients undergoing treatment [25]. In turn, the results obtained by Slovenian researchers indicated that delays in cancer diagnostics and treatment in some patients during the COVID-19 pandemic depended on factors attributable to doctors (lesser availability), patients (fear of infection—avoiding health care facilities) and the healthcare system and its management (exclusion of clinics and wards during the pandemic or allocating them exclusively to the COVID-19 treatment) [28].

## 5. Conclusions

Convincing patients about the proper preparation of health care for diagnostics and therapy is an important element influencing patient satisfaction with oncological care. This is particularly true during the pandemic.

Patient education and full implementation of preventive procedures during the pandemic reduces additional stress associated with the threat of infection. Minimizing stress is an important element in cancer treatment.

Delays in treatment (in particular in cancer treatment) have a negative impact on patient satisfaction with oncological care. Every effort should be made to maintain the timeliness of health services, in particular in oncology.

## Figures and Tables

**Table 1:** Patient characteristics in terms of sociodemographic and medical (clinical) features.

Characteristic					
Age	*N*	*M*	*Min.*	*Max.*	*SD*
	394	58.1	24.0	89.0	12.6
Sex	***N***		**%**		
female	225		57.1		
male	169		42.9		
Place of residence					
village	192		48.7		
town with up to 10,000 residents	67		17.0		
town with 10,000–50,000 residents	92		23.4		
city above 50,000 residents	43		10.9		
Marital status					
single	134		34.0		
in a relationship	260		66.0		
Education					
primary	38		9.6		
vocational	117		29.7		
secondary	151		38.3		
tertiary	88		22.4		
Financial situation					
very good or good	242		61.4		
sufficient or bad	152		38.6		
Number of children					
none	61		15.5		
one	50		12.7		
two	114		28.9		
three	99		25.2		
four and more	70		17.7		
Hospitalization due to					
treatment	338		85.6		
remission after treatment—follow-up examinations	56		14.4		
Type of diagnosed cancer					
breast cancer	86		21.8		
colorectal cancer	47		11.9		
prostate cancer	46		11.7		
myeloma	38		9.7		
ovarian cancer	36		9.1		
other	141		35.8		
Date of cancer diagnosis					
in 2020	110		27.9		
in 2019	111		28.2		
in 2018 and earlier	173		43.9		

*M*—arithmetic mean, *SD*—standard deviation, *Min*—minimum, *Max*—maximum.

**Table 2:** Patient satisfaction with safety procedures in the oncology hospital during the COVID-19 pandemic.

Question	Answer	*N*	%
What activities preceded your hospitalization? (multiple choice possible)	epidemiological interview	267	67.77
	temperature measurement	216	54.82
	conversation	104	26.40
	filling in a questionnaire on the possibility of exposure to contact with sick people	121	30.71
	health assessment	185	46.95
In your opinion, is the hospital adequately prepared for diagnostics and treatment of patients during the coronavirus pandemic (SARS-CoV-2)?	yes	339	86.04
	no	4	1.01
	I don’t have an opinion	51	12.95
During your hospitalization, were doctors and nurses equipped with appropriate personal protective equipment, such as masks and gloves?	yes	379	96.20
	no	2	0.51
Were the rules of the sanitary regime observed in places such as the waiting room, registration room, in particular a 2 m distance?	yes	301	76.40
	no	28	7.11
	sometimes	65	16.49
During your hospitalization, did you feel the risk of becoming infected with the SARS-CoV-2 virus?	yes	173	43.91
	no	221	56.09

**Table 3:** Assessment of the impact of the pandemic on the organization of cancer treatment.

Question	Answer	*N*	%
Were there any delays in cancer treatment due to the SARS-CoV-2 virus pandemic?	yes	110	27.92
	no	284	72.08
How long was the delay?	2 weeks	28	25.45
	>2 weeks to 1 month	35	31.82
	more than one month	47	42.73
Do you think that the SARS-CoV-2 virus pandemic disrupted cancer treatment and diagnostics?	yes	62	15.74
	no	163	41.37
	partially	169	42.89

**Table 4:** Patient satisfaction with oncological care—results for EORTC IN—PATSAT 32.

EORTC IN—PATSAT 32	Scale	*N*	*M*	*Min.*	*Max.*	*SD*
Competences of doctors	Technical skills	394	77.33	16.66	100.00	18.78
	Interpersonal skills		74.13	0.00	100.00	19.84
	Information provision		77.16	8.33	100.00	18.90
	Availability		75.60	12.50	100.00	20.89
Competences of nurses	Technical skills	394	79.86	25.00	100.00	17.42
	Interpersonal skills		79.34	8.33	100.00	17.54
	Information provision		79.59	25.00	100.00	16.82
	Availability		80.11	25.00	100.00	17.91
Other areas	Other hospital staff, interpersonal skills and availability of information		78.02	25.00	100.00	17.94
	Waiting time for medical procedures	394	76.07	12.50	100.00	19.73
	Hospital availability		69.83	12.50	100.00	22.25
	Information exchange		78.87	0.00	100.00	19.35
	Comfort		79.25	25.00	100.00	19.68
	General satisfaction		80.77	25.00	100.00	17.47

*M*—arithmetic mean, *SD*—standard deviation, *Min*—minimum, *Max*—maximum.

**Table 5:** Satisfaction with oncological care and the date of cancer diagnosis.

EORTC IN—PATSAT 32	Scale	Date of Cancer Diagnosis						*p*
		2020		2019		2018 and Earlier		
		*M*	*SD*	*M*	*SD*	*M*	*SD*	
Competences of doctors	Technical skills	73.64 ^a^	18.84	76.80 ^a,b^	19.59	80.68 ^b^	17.42	0.007
	Interpersonal skills	70.68	21.85	74.62	21.70	76.25	17.26	0.072
	Information provision	74.24	20.02	76.52	20.16	79.53	17.60	0.069
	Availability	70.91 ^a^	21.09	75.99 ^a,b^	23.07	79.48	18.18 ^b^	0.002 *
Competences of nurses	Technical skills	76.89 ^a^	18.29	79.64 ^a,b^	17.95	82.66 ^b^	16.21	0.023
	Interpersonal skills	76.14	19.04	80.30	16.72	80.78	17.12	0.082
	Information provision	75.38 ^a^	18.42	80.21 ^a,b^	17.38	82.42 ^b^	15.16	0.004 *
	Availability	75.91 ^a^	18.53	81.68 ^a,b^	17.97	83.16 ^b^	16.65	0.003
Other areas	Other hospital staff, interpersonal skills and availability of information	74.02 ^a^	18.12	77.84 ^a,b^	19.16	81.12	16.86 ^b^	0.004
	Waiting time for medical procedures	74.20	19.91	75.28	20.59	78.83	18.40	0.114
	Hospital availability	64.66 ^a^	23.45	73.01 ^b^	21.77	72.04 ^b,c^	22.78	0.008
	Information exchange	73.64 ^a^	20.55	79.55 ^a,b^	18.39	82.37 ^b^	18.28	0.001
	Comfort	75.68 ^a^	20.73	77.84 ^a,b^	19.85	82.37 ^b^	18.48	0.014
	General satisfaction	78.18	18.89	80.97	16.95	82.80	16.74	0.096

*M*—arithmetic mean, *SD*—standard deviation, *—Welch *p*; a, b, c—mean values in different letters differ statistically significantly at *p* < 0.05.

**Table 6:** Satisfaction with oncological care and delays in cancer treatment.

EORTC IN—PATSAT 32	Scale	Yes		No		*p*
		*M*	*SD*	*M*	*SD*	
Competences of doctors	Technical skills	72.35	20.57	79.25	17.71	0.001 *
	Interpersonal skills	68.48	22.98	76.32	18.05	0.000 *
	Information provision	73.11	21.97	78.73	17.36	0.008 *
	Availability	68.41	24.58	78.39	18.59	0.000 *
Competences of nurses	Technical skills	76.59	19.44	81.13	16.44	0.032
	Interpersonal skills	75.61	19.44	80.78	16.55	0.015
	Information provision	77.05	18.21	80.58	16.18	0.077
	Availability	76.36	19.27	81.56	17.18	0.014
Other areas	Other hospital staff, interpersonal skills and availability of information	75.00	19.91	79.20	17.01	0.053
	Waiting time for medical procedures	72.16	22.06	77.60	18.57	0.014 *
	Hospital availability	66.25	23.98	71.25	21.43	0.059
	Information exchange	74.32	21.80	80.63	18.05	0.008
	Comfort/cleanliness	79.55	21.20	79.14	19.10	0.860
	General satisfaction	76.82	19.07	82.31	16.60	0.009

*M*—arithmetic mean, *SD*—standard deviation, *—Cochran-Coxa *p*.

**Table 7:** Patient satisfaction with oncological care, taking into account the opinion that the COVID-19 pandemic disrupted cancer diagnostics and treatment.

EORTC IN—PATSAT 32	Scale	Disorganization of Cancer Diagnostics and Treatment during the COVID-19 Pandemic						*p*
		Yes		No		Partially		
		*M*	*SD*	*M*	*SD*	*M*	*SD*	
Competences of doctors	Technical skills	68.95 ^a,c^	21.61	75.36^c^	17.60	82.30 ^b^	17.38	0.000 *
	Interpersonal skills	65.46 ^a^	25.24	74.90 ^b,c^	16.82	76.58 ^c^	19.54	0.008 *
	Information provision	69.89 ^a^	24.58	76.18 ^a,b^	18.02	80.77 ^b^	16.42	0.002 *
	Availability	67.94 ^a,c^	26.46	73.93 ^c^	18.60	80.03 ^b^	19.73	0.001 *
Competences of nurses	Technical skills	75.94 ^a,c^	19.39	76.89 ^c^	16.42	84.17 ^b^	16.76	0.000
	Interpersonal skills	74.33 ^a,c^	21.73	77.25 ^c^	15.69	83.19 ^b^	16.82	0.000
	Information provision	75.67 ^a,c^	20.15	78.27 ^c^	15.67	82.30 ^b^	16.22	0.012
	Availability	75.81 ^a,c^	19.72	78.91 ^c^	16.17	82.84 ^b^	18.49	0.026 *
Other areas	Other hospital staff, interpersonal skills and availability of information	72.72 ^a,c^	20.86	75.15 ^c^	17.01	82.74 ^b^	16.58	0.000
	Waiting time for medical procedures	67.94 ^a,c^	23.81	74.08 ^c^	18.61	80.99 ^b^	17.84	0.000 *
	Hospital availability	64.52 ^a,c^	25.83	68.25 ^c^	21.66	73.30 ^b^	20.95	0.020 *
	Information exchange	70.97 ^a,c^	23.61	75.92 ^c^	16.64	84.62 ^b^	18.50	0.000 *
	Comfort/cleanliness	76.61 ^a,c^	22.17	76.07 ^c^	18.08	83.28 ^b^	19.62	0.002 *
	General satisfaction	75.81 ^a,c^	19.72	79.14 ^c^	16.96	84.17 ^b^	16.51	0.002

*M*—arithmetic mean, *SD*—standard deviation, *—Welch *p*; a, b, c—mean values in different letters differ statistically significantly at *p* < 0.05.

## Appendix A

### The Questionnaire Developed by the Authors

1. Age
2. Sex
  - female
  - male
3. Place of residence
  - village
  - town with up to 10,000 residents
  - town with 10,000–50,000 residents
  - city above 50,000 residents
4. Marital status
  - single
  - in a relationship
5. Education
  - primary
  - vocational
  - secondary
  - tertiary
6. Financial situation
  - very good or good
  - sufficient or bad
7. Number of children
  - none
  - one
  - two
  - three
  - four and more
8. Hospitalization due to:
  - treatment
  - remission after treatment—follow-up examinations
9. Type of diagnosed cancer
  - breast cancer
  - colorectal cancer
  - prostate cancer
  - myeloma
  - ovarian cancer
  - other
10. Date of cancer diagnosis
  - in 2020
  - in 2019
  - in 2018 and earlier
11. What activities preceded your hospitalization? (multiple choice possible)
  - epidemiological interview
  - temperature measurement
  - conversation
  - filling in a questionnaire on the possibility of exposure to contact with sick people
12. In your opinion, is the hospital adequately prepared for diagnostics and treatment of patients during the coronavirus pandemic (SARS-CoV-2)?
  - yes
  - no
  - I don’t have an opinion
13. During your hospitalization, were doctors and nurses equipped with appropriate personal protective equipment, such as masks and gloves?
  - yes
  - no
14. Were the rules of the sanitary regime observed in places such as the waiting room, registration room, in particular a 2 m distance?
  - yes
  - no
  - sometimes
15. During your hospitalization, did you feel the risk of becoming infected with the SARS-CoV-2 virus?
  - yes
  - no
16. Were there any delays in cancer treatment due to the SARS-CoV-2 virus pandemic?
  - yes
  - no
17. How long was the delay?
  - 2 weeks
  - >2 weeks to 1 month
  - more than one month
18. Do you think that the SARS-CoV-2 virus pandemic disrupted cancer treatment and diagnostics?
  - yes
  - no
  - partially
